# Effects of interpersonal sensitivity on depressive symptoms in postgraduate students during the COVID-19 pandemic: Psychological capital and sleep quality as mediators

**DOI:** 10.3389/fpsyt.2023.1100355

**Published:** 2023-04-06

**Authors:** Xin Liu, Lixin Peng, Zhen Wang, Ping Zeng, Yanyan Mi, Haibo Xu

**Affiliations:** ^1^Center for Mental Health Education and Research, Xuzhou Medical University, Xuzhou, China; ^2^School of Management, Xuzhou Medical University, Xuzhou, China; ^3^Department of Epidemiology and Biostatistics, School of Public Health, Xuzhou Medical University, Xuzhou, China

**Keywords:** interpersonal sensitivity, depressive symptoms, psychological capital, sleep quality, COVID-19 pandemic

## Abstract

**Background:**

This study aimed to examine depressive symptoms associated with interpersonal sensitivity, sleep quality, and psychological capital among postgraduate students during static campus management after the COVID-19 pandemic in China.

**Methods:**

Research data were obtained during static campus management (10–19 April 2022) after the reappearance of COVID-19 in cities in eastern China. We collected data through an online questionnaire, and the anonymous self-reported questionnaire included the Patient Health Questionnaire, the interpersonal sensitivity subscale of Symptom Checklist-90, the Psychological Capital Questionnaire, and the Pittsburgh Sleep Quality Index. analysis of variance was performed using *t*-test and ANOVA. The PROCESS macro was used to determine the relationship between interpersonal sensitivity and depression, together with the independent and serial mediating role of psychological capital and sleep quality.

**Results:**

A total of 2,554 postgraduate students were included in this study. The prevalence of mild, moderate, and severe depressive symptoms was 30.97, 6.58, and 1.45%, respectively. Interpersonal sensitivity was significantly associated with depressive symptoms (direct effect = 0.183, *p* < 0.001). Between interpersonal sensitivity and depressive symptoms, psychological capital and sleep quality played a single mediating role (indirect effect = 0.136 and 0.100, *p* < 0.001, respectively) and a chain mediating role together (indirect effect = 0.066, *p* < 0.001).

**Conclusion:**

Interpersonal sensitivity has a significant influence on depression among Chinese graduate students. Psychological capital and sleep quality may not only independently mediate the relationship between interpersonal sensitivity and depression, but also co-play a chain-mediating role in the pathway from interpersonal sensitivity to depression. Positive psychological interventions and sleep guidance may be beneficial in alleviating depressive symptoms.

## Introduction

To control the rapid spread of COVID-19, the World Health Organization (WHO) has recommended a series of measures. One of those measures, lockdown, has proven effective in controlling the spread of the disease (1, 2). The prolonged lockdown has brought the epidemic under control to a certain extent, but it has also had a detrimental effect on people’s physical and mental health. Recent research has shown that mental health problems were more severe in the general population during the lockdown period (3). For example, children’s mental health worsened as a result of the pandemic in England (4), and adults experienced worse psychological outcomes during the pandemic in Saudi Arabia (5). In addition, the lockdown measures had a negative impact on the mental health of adolescents (6) and the elderly (7). Depression was found to be one of the most common and prominent psychological symptoms during this period (8). Almost half of the adult participants reported depressive symptoms during the lockdown (9), and the prevalence of depression in the general population (70.10%) was significantly higher compared with COVID-19 patients (39.50%) (10).

Locking down university campuses is a static management method used in China. In order to protect students from the COVID-19 pandemic, all college students are not allowed to enter and leave the campus gates at will during static management, but they are allowed to carry out activities on campus according to their actual needs, such as walking, eating, and shopping for daily necessities. It was found that the mental health of college students had been affected by campus lockdown (11), more so than administrative staff (12). Longitudinal analyses have shown a significant increase in depressive symptoms among college students during lockdown (13, 14). Compared with undergraduate students, postgraduate students typically face more pressure from scientific research and study (15), especially given the shortage of tutoring during the COVID-19 pandemic, which could lead them to experience anxiety or depression. A survey of 3137 Chinese postgraduate students showed that the prevalence of depressive symptoms was as high as 33.87% (16). One study agrees that women are more likely to suffer from depression than men (17). However, a study in China found that a significantly higher percentage of male students suffered from depression than their female counterparts (17). A Norwegian study of medical students found no gender differences in mental health or stress during medical school (18).

In addition to depression, sleep was also affected during the lockdown (19). Sleep quality includes quantitative aspects of sleep, such as sleep duration, sleep latency, or the number of awakenings, but also more purely subjective aspects, such as the “depth” or “restfulness” of sleep (20). Studies have found that sleep duration increased and sleep quality paradoxically decreased in college students (21, 22). Meanwhile, depressive symptoms were highly correlated with poor sleep quality among college students (13). A survey found that sleep quality was an important predictor of an individual’s mental wellbeing (23). Furthermore, sleep quality may mediate the relationship between negative emotions or behaviors and depression among college students, such as academic stress (24) or Internet addiction (25). However, research is scarce on the sleep quality of postgraduate students during the lockdown.

Interpersonal sensitivity itself is a prominent mental health problem faced by contemporary college students (26), reflecting the quality of individual interpersonal interaction. Sometimes major traumatic events can amplify the negative aspects of interpersonal relationships (27), especially the limited social contact leads to a lack of social support (28) during the COVID-19 pandemic, although online media can support online interaction, the effect is not as good as face-to-face interaction (29). A study in Morocco found that individuals showed significantly higher levels of interpersonal sensitivity during COVID-19 (30). Furthermore, interpersonal sensitivity can trigger or exacerbate psychiatric symptoms (31), such as anxiety (32), depression (33), and so on. According to the diathesis-stress model, an individual’s own personality vulnerability is stimulated by external stressful events, leading to a sustained increase in depression, and when the external stimulus reaches a certain level, the individual becomes depressed (34). In addition, interpersonal problems may directly affect sleep quality in college students (35). One study showed that real-life interpersonal interaction was positively associated with sleep quality and may improve it (36). Nonetheless, in the case of limited face-to-face interpersonal interaction, there is little research on the mediating role of sleep quality in the relationship between interpersonal sensitivity and depression among Chinese postgraduate students.

Psychological capital (PsyCap) has been defined as the positive psychological state that individuals show in the process of their own growth and development, including four components: self-efficacy, optimism, hope, and resilience (37). Previous studies demonstrated that PsyCap was negatively correlated with depression, and can ease depressive symptoms (38) and reduce negative emotions or prevent psychological symptoms (39). In addition, PsyCap may mediate the relationship between stress or work-family conflict and depression (40, 41). However, the role of PsyCap in the pathway from interpersonal sensitivity to depression remains unclear. In addition, PsyCap has been shown to be a robust predictor of sleep quality (42). PsyCap may also mediate the effects of dysfunctional sleep beliefs on wellbeing (43) and the pathway from organizational justice variability to employee sleep quality (44). Currently, there is little research on the sleep quality of postgraduate students during the lockdown.

**FIGURE 1:**
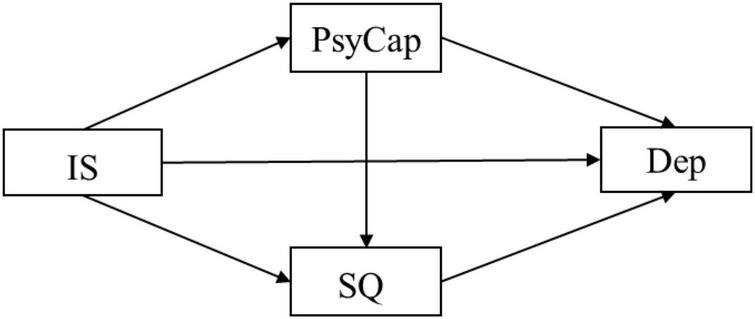
The chained mediation models. In this figure, the independent variable is interpersonal sensitivity (IS), the dependent variable is depression (Dep), and the mediators are psychological capital (PsyCap) and sleep quality (SQ).

In conclusion, this study examines interpersonal sensitivity, psychological capital, sleep quality, and depression to investigate the mental health of graduate students in the context of COVID-19 and campus lockdown, providing guidance for university mental health departments to deal with depression caused by COVID-19 and other pandemic diseases. Thus, our study further explores the mediating role of psychological capital and sleep quality on the pathway from interpersonal sensitivity to depressive symptoms, which are listed below along with a multiple mediation model (Figure 1):

**Hypothesis 1:** interpersonal sensitivity has a significant impact on depression among Chinese postgraduate students;

**Hypothesis 2:** PsyCap and sleep quality can independently mediate the relationship between interpersonal sensitivity and depression;

**Hypothesis 3:** PsyCap and sleep quality co-play a cascading mediating role in the pathway from interpersonal sensitivity to depression.

## Materials and methods

### Participants and procedure

The research sample data were collected from the Student Mental Health Center at a university in eastern China, 2 weeks into its campus static management (10–19 April 2022) after the reappearance of COVID-19 in the city in 2022. All participants completed the questionnaire by scanning a QR code and then filling it out. Inclusion criteria: postgraduate students enrolled in the target university; postgraduate students who volunteered to participate in the survey after reading the instructions.

Exclusion criteria: in principle, there are no exclusion criteria since the online questionnaire platform used for this research sets the restriction that the questionnaire cannot be submitted until the questions are answered. During data screening, researchers excluded the questionnaires that: (1) had obvious logical errors; (2) had a clear pattern of responses.

This study was approved by the Ethics Committee of Xuzhou Medical University. All procedures were carried out in accordance with relevant ethical guidelines and regulations.

### Measurements

The survey consisted of two sections. First, the social, demographic, and other relevant characteristics of the postgraduate students were recorded *via* the questionnaire, including gender, age, grade, region, whether being an only child, whether living with parents during childhood, and whether having concerns about COVID-19, eating breakfast, and exercising or not. Second, the survey included four scales as the assessment tools (see below for details):

#### Depressive symptoms

The 9-item Patient Health Questionnaire Depression Scale (PHQ-9) was used to measure the severity of depressive symptoms. The PHQ-9 is the most widely used depression measure in the world and has shown good reliability and validity in the context of the pandemic (45). The scale consists of nine items, with each item rated from 0 (“never”) to 3 (“almost every day”), and the total scores of 5, 10, 15, and 20 represent the thresholds for mild, moderate, moderately severe, and severe depressive symptoms, respectively. The PHQ-9 has been shown to have acceptable psychometric properties for screening depression among Chinese undergraduate students (46). In this study, Cronbach’s alpha for the scale was 0.913.

#### Interpersonal sensitivity

The interpersonal sensitivity subscale of Symptom Checklist 90 (SCL-90) was used (47), which has been used among Chinese college students and has shown good reliability (48). The subscale consists of 9 items, and each of these is rated from 0 (“none”) to 4 (“very severe”). A higher total score of all items indicates a higher level of interpersonal sensitivity. In this study, Cronbach’s alpha for the scale was 0.921.

#### Psychological capital

The psychological capital of college students was measured by the Psychological Capital Questionnaire (PCQ-24), which was developed by Luthans et al. (37), and the Chinese version has shown good reliability (49). The scale consists of four dimensions and 24 items. Each item is scored on a 6-point Likert scale, with 1 indicating “strong disagreement” and 6 indicating “strong agreement.” A higher score generally indicates a higher level of psychological capital. In this study, Cronbach’s alpha for the PCQ-24 was 0.952.

#### Sleep quality

The Pittsburgh Sleep Quality Index (PSQI) was adopted to measure the sleep quality of individuals. The scale was developed by Buysse et al. (50) and includes seven components so that each dimension has four items. The sum score of all items was the sleep quality index. The higher the score, the worse the quality of the individual’s sleep. In addition, a total score > 5 was indicative of poor sleep quality. It has been demonstrated to be a reliable and valid method for assessing and screening sleep dysfunctions among Chinese college students (51). In our study, Cronbach’s alpha for PSQI was 0.743.

### Statistical analysis

Mean and standard deviation was used to describe the basic information of the data. Pearson’s coefficient was used to show the correlation between the four variables (interpersonal sensitivity, depression, PsyCap, and sleep quality). Independent samples *t*-test and one-way analysis of variance (ANOVA) were employed to describe the distribution of the four variables. The PROCESS v 3.4 macro was used to test the serial multiple mediation model, and models 4 and 6 were selected (52). All of the above analyses were completed with SPSS 22.0, and all significance tests were two-tailed, α = 0.05.

## Results

### Subjects and sample selection

We adopted G*Power 3.1.9.2 to calculate the sample size. A *post-hoc* analysis in G*Power was used to calculate the achieved power (1-β) of the sample size of our study. Since the process macro was based on the multiple regression model, the fixed model of linear multiple regression was set as the statistical test. The calculated parameters were as follows: the effect size f2 was entered as 0.15, α was 0.05, the total sample was 2554, and the number of final predictors was 12 (the number of tested predictors was 3). The result showed that the statistical power of the regression model was 100%, indicating that 2554 postgraduate students reached the necessary sample size.

### Preliminary analyses

A total of 2,554 postgraduate students participated in the study (mean_*year*_ = 25.85, standard deviation = 2.63), with 36.53% of male participants and 63.47% of female participants (Table 1).

**TABLE 1:** Sociodemographic characteristics of four variables among postgraduate students.

Category	*N* (%)	Depression		Interpersonal sensitivity		Psychological capital		Sleep quality	
		**M ± SD**	***t/F (p)***	**M ± SD**	***t/F (p)***	**M ± SD**	***t/F (p)***	**M ± SD**	***t/F (p)***
**Total**	2554	4.51 ± 4.69		1.91 ± 0.68		104.92 ± 16.39		4.69 ± 3.03	
**Gender**									
Male students	933 (36.5)	4.57 ± 5.16	*t* = 0.459 (*p* = 0.647)	1.95 ± 0.74	*t* = 1.765 (*p* = 0.078)	105.93 ± 17.22	*t* = 2.301 (*p* = 0.021)	4.54 ± 3.28	*t* = −1.782 (*p* = 0.075)
Female students	1621 (63.5)	4.48 ± 4.39		1.89 ± 0.64		104.34 ± 15.86		4.77 ± 2.88	
**Age**									
22–25	1361 (53.3)	4.43 ± 4.62	*F* = 0.472 (*p* = 0.624)	1.91 ± 0.69	*F* = 2.321 (*p* = 0.098)	105.01 ± 16.71	*F* = 0.198 (*p* = 0.820)	4.47 ± 2.88	*F* = 9.745 (*p* < 0.001)
26–30	1043 (40.8)	4.61 ± 4.74		1.90 ± 0.66		104.92 ± 15.89		4.86 ± 3.11	
>30	150 (5.9)	4.60 ± 4.94		2.03 ± 0.66		104.13 ± 16.92		5.43 ± 3.63	
**Grade**									
First	971 (38.0)	4.12 ± 4.45	*F* = 7.620 (*p* = 0.001)	1.89 ± 0.69	*F* = 0.661 (*p* = 0.516)	106.14 ± 16.14	*F* = 4.927 (*p* = 0.007)	4.38 ± 2.87	*F* = 8.917 (*p* < 0.001)
Second	914 (35.8)	4.96 ± 4.90		1.93 ± 0.68		103.79 ± 16.88		4.78 ± 3.01	
Third	669 (26.2)	4.48 ± 4.68		1.92 ± 0.66		104.70 ± 15.95		5.01 ± 3.26	
**Region**									
Rural	1474 (57.7)	4.66 ± 4.76	*t* = 1.788 (*p* = 0.074)	1.94 ± 0.68	*t* = 2.397 (*p* = 0.017)	104.26 ± 16.10	*t* = −2.378 (*p* = 0.017)	4.68 ± 2.93	*t* = −0.164 (*p* = 0.869)
Urban	1080 (42.3)	4.32 ± 4.58		1.88 ± 0.68		105.82 ± 16.74		4.70 ± 3.17	
**Only child**									
Yes	949 (37.2)	4.32 ± 4.57	*t* = −1.597 (*p* = 0.110)	1.89 ± 0.70	*t* = −1.409 (*p* = 0.159)	106.32 ± 16.40	*t* = 3.322 (*p* = 0.001)	4.60 ± 3.19	*t* = −1.100 (*p* = 0.271)
No	1605 (62.8)	4.63 ± 4.75		1.93 ± 0.66		104.10 ± 16.32		4.74 ± 2.94	
**Living w/parents as a child**									
Yes	2228 (87.2)	4.39 ± 4.61	*t* = −3.567 (*p* < 0.001)	1.89 ± 0.68	*t* = −4.863 (*p* < 0.001)	105.44 ± 16.44	*t* = 4.189 (*p* < 0.001)	4.61 ± 2.97	*t* = −3.201 (*p* = 0.001)
No	326 (12.8)	5.38 ± 5.11		2.08 ± 0.67		101.38 ± 15.60		5.24 ± 3.38	
**Concerned about Covid-19**									
Yes	2438 (95.5)	4.41 ± 4.55	*t* = −3.653 (*p* < 0.001)	1.90 ± 0.66	*t* = −4.551 (*p* < 0.001)	105.39 ± 16.07	*t* = 5.570 (*p* < 0.001)	4.63 ± 3.01	*t* = −4.265 (*p* < 0.001)
No	116 (4.5)	6.66 ± 6.56		2.29 ± 0.91		95.09 ± 19.61		5.97 ± 3.34	
**Eating breakfast**									
No	474 (18.6)	5.99 ± 5.39	*F* = 45.743 (*p* < 0.001)	2.05 ± 0.73	*F* = 23.903 (*p* < 0.001)	99.72 ± 17.22	*F* = 50.726 (*p* < 0.001)	5.68 ± 3.40	*F* = 45.778 (*p* < 0.001)
Seldom	954 (37.4)	4.80 ± 4.71		1.96 ± 0.67		103.67 ± 15.58		4.83 ± 3.02	
Usually	1126 (44.0)	3.65 ± 4.13		1.82 ± 0.65		108.17 ± 15.99		4.15 ± 2.75	
**Physical exercise**									
No	459 (17.9)	5.47 ± 5.19	*F* = 15.709 (*p* < 0.001)	2.07 ± 0.73	*F* = 22.542 (*p* < 0.001)	98.95 ± 16.78	*F* = 52.114 (*p* < 0.001)	5.51 ± 3.24	*F* = 29.576 (*p* < 0.001)
Seldom	1340 (52.5)	4.53 ± 4.58		1.92 ± 0.66		104.86 ± 15.67		4.71 ± 2.98	
Usually	756 (29.6)	3.92 ± 4.45		1.81 ± 0.66		108.66 ± 16.32		4.15 ± 2.89	

In our study, the mean score of the PHQ-9 was 4.51, and the prevalence of mild, moderate, and severe depressive symptoms was 30.97, 6.58, and 1.45%, respectively. According to the PSQI threshold, the proportion of participants with poor sleep quality was 35.40%. The average score of interpersonal sensitivity and PsyCap were 1.91 and 104.92, respectively. Interestingly, there was a significant difference in the four variables among all postgraduate students who lived with parents during childhood (*p* < 0.001), were concerned about COVID-19 (*p* < 0.001), ate breakfast (*p* < 0.001), and exercised (*p* < 0.001). Specifically, the postgraduate students who did not live with parents when they were between 0 and 6 years old, were not concerned about COVID-19, did not eat breakfast, and did not exercise had higher scores on interpersonal sensitivity, sleep quality, and depression, and lower scores on PsyCap.

### Correlation analysis

Correlations for all variables are presented in Table 2. Depression was positively related to interpersonal sensitivity and sleep quality (*r* = 0.508 and 0.593, *p* < 0.001, respectively) and negatively related to PsyCap (*r* = −0.546, *p* < 0.001). Interpersonal sensitivity was also positively related to sleep quality (*r* = 0.459, *p* < 0.001). In addition, a negative correlation was identified between PsyCap and sleep quality (*r* = −0.492, *p* < 0.001).

**TABLE 2:** Pearson’s correlation between variables.

	Dep	IS	PsyCap	SQ
Depressive symptoms (Dep)	–			
Interpersonal sensitivity (IS)	0.508***	–		
Psychological capital (PsyCap)	−0.546***	−0.579***	–	
Sleep quality (SQ)	0.593***	0.459***	−0.492***	–

***p < 0.001.

### Mediation analysis

**FIGURE 2:**
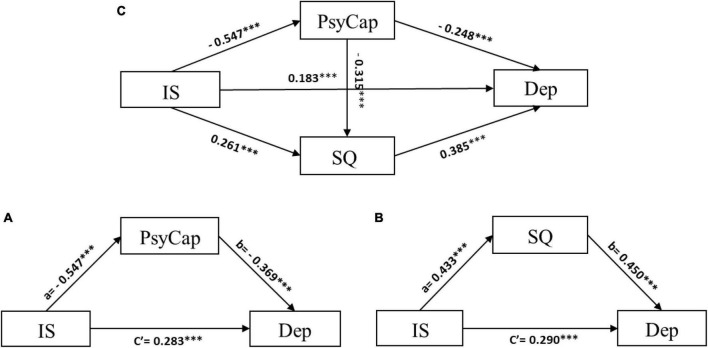
The characteristics of models. In this figure, psychological capital (PsyCap) as an independent mediator of the model **(A)**; sleep quality (SQ) as an independent mediator of the model **(B)**; psychological capital (PsyCap) and sleep quality (SQ) jointly play a mediating role of the chain mediation model **(C)**. ^***^p < 0.001.

As an essential first step, we tested the mediating role of PsyCap and sleep quality in the relationship between interpersonal sensitivity and depression, respectively. In the model with PsyCap as the mediator, interpersonal sensitivity had a negative association with PsyCap (*a* = −0.547, *p* < 0.001). With the statistically significant effect of PsyCap on depression (*b* = −0.369, *p* < 0.001), it appears that interpersonal sensitivity had a positive direct effect (*c’* = 0.283, *p* < 0.001), and further had an indirect effect on depression *via* partially mediating the role of PsyCap (indirect effect = 0.202, 95% bootstrap CI: 0.169–0.235) (Figure 2A).

In the model with sleep quality as a mediator, depression was positively affected by interpersonal sensitivity (*c’* = 0.290, *p* < 0.001) and sleep quality (*b* = 0.450, *p* < 0.001). In addition, sleep quality was also positively associated with interpersonal sensitivity (*a* = 0.433, *p* < 0.001). A significant indirect effect of interpersonal sensitivity on depression *via* sleep quality was observed (indirect effect = 0.195, 95% bootstrap CI: 0.169–0.222) (Figure 2B). H1 and H2 were supported by these findings.

After the single mediation model was tested, we conducted the serial multiple mediation model to verify H3. As shown in Figure 2C and Table 3 interpersonal sensitivity had a direct effect on depression (β = 0.183, *p* < 0.001). In addition, interpersonal sensitivity had a significant effect on PsyCap and sleep quality (β = −0.547 and 0.261, *p* < 0.001, respectively). Depression was impacted by PsyCap and sleep quality (β = −0.248 and 0.385, *p* < 0.001, respectively). As the basis of the serial model, a significant regression result was observed between PsyCap and sleep quality (β = −0.315, *p* < 0.001).

**TABLE 3:** Model characteristics of the serial multiple mediation models.

	Model 1 (*Y* = PsyCap)		Model 2 (*Y* = SQ)		Model 3 (*Y* = Dep)	
	**B**	***t***	**B**	***t***	**B**	***t***
(Constant)	0.155	0.941	−0.233	−1.348	0.161	1.056
Gender	−0.121	−3.603***	0.086	2.434*	−0.057	−1.812
Age	0.008	0.265	0.123	3.996***	−0.037	−1.365
Grade	−0.031	−1.406	0.035	1.511	0.003	0.154
Region	−0.012	−0.346	0.063	1.722	−0.032	−1.019
Only child	−0.086	−2.396*	−0.012	−0.305	0.009	0.276
Living w/Parents as a child	−0.032	−0.665	0.037	0.728	0.013	0.294
Concerned about COVID-19	−0.270	−3.540***	0.073	0.902	0.036	0.510
Eating breakfast	0.130	5.936***	−0.114	−4.923***	−0.061	−2.979**
Physical exercise	0.129	5.356***	−0.043	−1.673	0.047	2.102*
IS	−0.547	−33.844***	0.261	12.763***	0.183	9.853***
PsyCap			−0.315	−15.096***	−0.248	−12.954***
SQ					0.385	22.038***

*p < 0.05; **p < 0.01; ***p < 0.001.

IS, interpersonal sensitivity; PsyCap, psychological capital; SQ, sleep quality; Dep, depressive symptoms.

Table 4 shows the effect of the pathways of the serial model. In addition to the direct effect (β = 0.183, 95% bootstrap CI: 0.147–0.219), interpersonal sensitivity also had an indirect effect on depression *via* PsyCap and sleep quality (total indirect effect = 0.302, 95% bootstrap CI: 0.267–0.339). In the pathway *via* PsyCap only, the indirect effect was 0.136 (95% bootstrap CI: 0.108–0.166); and in the pathway *via* sleep quality only, the indirect effect was 0.100 (95% bootstrap CI: 0.080–0.122). In support of H3, the specific indirect effect of interpersonal sensitivity on depression through both mediators in the serial pathway (PsyCap-sleep quality) is also significant (serial indirect effect = 0.066, 95% bootstrap CI: 0.053–0.080). Based on the above results, our three hypotheses are all supported.

**TABLE 4:** The direct and indirect effect of the serial multiple models.

Pathway	Effect	SE	Bootstrap 95% CI	
			**LLCI**	**ULCI**
Direct	0.183	0.019	0.147	0.219
**Indirect**				
Total	0.302	0.018	0.267	0.339
ind1 (IS → PsyCap → Dep)	0.136	0.015	0.108	0.166
ind2 (IS → SQ → Dep)	0.100	0.011	0.080	0.122
ind3 (IS → PsyCap → SQ → Dep)	0.066	0.007	0.053	0.080

IS, interpersonal sensitivity; PsyCap, psychological capital; SQ, sleep quality; Dep, depressive symptoms.

## Discussion

This study was conducted to explore the *status quo* of depression and the mediating role of PsyCap and sleep quality on the relationship between interpersonal sensitivity and depression among Chinese postgraduate students. We found a high self-reported prevalence of depression among postgraduate students using the validated self-report questionnaire during the lockdown period, which was generally higher than the result (33.87%) reported by Liang (16) in the same population. There are two possible reasons for this difference: the diversity between the two measurement tools (53) and the time points of the lockdown. The tool used to measure depression in the survey from which we obtained data was the PHQ-9, and Liang used the SDS (Self-rating Depression Scale). This survey was conducted during a lockdown in 2022, which was repeated due to the renewed outbreak of COVID-19. Once again, normal college life was disrupted by the lockdown, which may cause more depressive symptoms. In addition, it was found that 35.40% of the participants in the study had poor sleep quality, which is comparable to the findings in Cremasco’s study (54). The research by Cremasco included 2838 students and revealed that more than 45% of participants reported a decline in sleep quality. In China, postgraduate students need to meet certain academic requirements before graduation. In the context of the lockdown, students were unable to pursue their own research projects as usual. The concern about their graduation and further education resulted in longer sleep but poorer sleep quality, which was consistent with the previous study (21).

It was observed that the score of depression had a significant difference in postgraduate students with different options for grades, whether they lived with their parents when they were 0–6, concerning information, eating breakfast, and doing physical activity. In China, the postgraduate education system is three years, and academic research begins in the second year generally. since then they face the great academic pressure because they need to read literatures or do experiments, and publish academic papers. Postgraduate students who did not live with their parents between ages 0 and 6 showed higher depression scores. We speculated that this might be related to parent-child attachment in childhood, and the study showed that insecure attachment increased the risk of depression (55). The insecurity of this attachment was magnified by the long-term lockdown, as the respondents were unable to meet with family members. Timely attention to the information related to the epidemic can give individuals a general understanding of epidemic prevention and control and reduce unnecessary panic and anxiety (56). It has been found that the risk of depressive symptoms tends to increase with decreasing frequency of eating breakfast (57), and skipping breakfast was a risk factor for eating disorders which was related to depressive symptoms (58). Meanwhile, those who usually exercised had the lowest depression scores. A meta-analysis showed that physical activity has significant mental health benefits (59). In addition, a randomized experimental trial showed that a physical activity intervention was an effective approach to depression in college students during the pandemic (60). Adequate exercise can alleviate depression caused by the epidemic, whether it is to physically improve immunity (61) or to psychologically feel safe.

Interestingly, we also found that the scores of the other three model variables (interpersonal sensitivity, PsyCap, and sleep quality) had the same differential changes as depression in these students with different choices about having lived with parents in childhood, who were concerned about information on COVID-19, having breakfast and exercising. In general, postgraduate students who did not live with their parents in childhood, were not concerned about the epidemic, did not have breakfast, or did not exercise had the poorest mental health. Postgraduate students should pay attention to these aspects and make changes in these aspects to ease psychological symptoms.

Central to our research findings is the examination of PsyCap and sleep quality are postulated as jointly mediating variables in a model of the relationship between interpersonal sensitivity and depression. Our findings demonstrated that the serial multiple mediation effect of PsyCap and sleep quality in sequence and the separate mediation effect of them were both significant. The results also provided new insights into the differences in the strength of the indirect effect of PsyCap and sleep quality on this association. Specifically, it was found that the serial indirect effect of interpersonal sensitivity on depression through PsyCap was found to be stronger than the other two indirect effect pathways.

Our research found that interpersonal sensitivity has a significant impact on depression among Chinese postgraduate students. Some of the manifestations of interpersonal sensitivity are highly consistent with depression, especially characteristics such as sensitivity and suspiciousness, and low self-esteem (62). People with higher levels of interpersonal sensitivity do less well in maintaining long-term relationships; they may have few friends and always prefer to keep to themselves, leading to increased loneliness and eventually depression. Negative core beliefs about the self were central to the development of depression (63). A meta-analysis revealed that the COVID-19 lockdowns, which were characterized by isolation and reduced social contact, played a significant role in the increased negative emotional symptoms of college students (64). In addition, supervisors should provide timely advice related to the research and allow postgraduate students to present their work, which can develop students’ skills (65). However, online contact weakened the communication effect between supervisors and students, which made students unable to receive timely feedback on their current work, which in turn, led to increased negative emotions. The negative emotional symptoms amplified the effect of interpersonal sensitivity on postgraduate students’ depression. Furthermore, a long period in a negative state may weaken an individual’s positive state, as shown by the results of research conducted during school lockdowns, which found that the resilience in Chinese adolescents decreased due to the closures (66). High levels of interpersonal sensitivity may result in postgraduate students having worse interpersonal relationships, which may not be conducive to the expansion of their resources and may affect the development of their psychological capital, and further lead to depression. Therefore, as a high-order positive psychological variable, PsyCap mediated the relationship between interpersonal sensitivity and depression during the lockdown.

Furthermore, sleep quality also independently played a mediating role in the relationship, which was consistent with previous research (25). Studies have indicated that interpersonal problems can directly affect sleep quality (37), and more interpersonal stress was associated with more cognitive pre-sleep arousal (67). We infer that interpersonal sensitivity of graduate students may lead to negative emotional symptoms, which may lead to poor sleep quality. Daily negative emotion was reported to be associated with greater sleep problems (68) and to mediate the effect of spontaneous brain activity on sleep quality (69). Meanwhile, sleep quality was associated with depression (70). Evidence suggests that sleep is an important biological mechanism in mood regulation (71) and that repeated sleep disruption may have direct effects on recovery and health.

Our findings also revealed the serial multiple mediating roles of PsyCap and sleep quality in the pathway from interpersonal sensitivity to depression in Chinese postgraduate students. Sleep quality was negatively affected by PsyCap in our study, which was consistent with previous research in different populations (42, 43). The psychological capital of postgraduate students may be affected to varying degrees by interpersonal sensitivity, which may affect the quality of sleep through psychological capital, leading to depression. The significant result further confirmed the rationality of our hypothesis. Hence, university mental health departments should pay attention to the related factors (self-efficacy, hope, resilience, optimism, etc.) that can improve the PsyCap of postgraduate students, provide appropriate mental health education in peacetime and conduct relevant psychological training for the groups with low PsyCap. At the same time, postgraduate students should also be provided with sleep knowledge and health education to make them aware of the importance of adequate sleep.

Several limitations of our research should be acknowledged. First, due to the nature of the cross-sectional study, the causal relationship of the mediation model was not sufficiently effective. A longitudinal study method should be adopted to test the causal relationship. Second, our study only included postgraduate students from only one university, which limited the generalizability of our findings. The scope of the study should be expanded. Third, because the main aim of this study was to investigate an intervention mechanism, the effect of covariates on depression was not examined, which may have led to biased results.

## Conclusion

This study provided empirical evidence on the pathways linking interpersonal sensitivity and depression among Chinese postgraduate students. The results showed that interpersonal sensitivity was significantly associated with depressive symptoms. Psychological capital and sleep quality played independent and cumulative mediating roles in the relationship between interpersonal sensitivity and depression. In addition, the single mediation model indicated that psychological capital had a greater impact than sleep quality on the relationship between interpersonal sensitivity and depression. The finding has important implications for the importance of positive psychological factors concerning the mental health of postgraduate students during the lockdown. Positive psychological interventions and sleep guidance should be considered to alleviate depressive symptoms in postgraduate students.

## Data availability statement

The original contributions presented in this study are included in the article/supplementary material, further inquiries can be directed to the corresponding author.

## Ethics statement

This studies was involving human participants were reviewed and approved by the Xuzhou Medical University Ethics Committee. The patients/participants provided their written informed consent to participate in this study.

## Author contributions

All authors listed have made a substantial, direct, and intellectual contribution to the work, and approved it for publication.
